# Fanconi anemia with sun-sensitivity caused by a Xeroderma pigmentosum-associated missense mutation in *XPF*

**DOI:** 10.1186/s12881-018-0520-1

**Published:** 2018-01-11

**Authors:** Isabell Popp, Maqsood Punekar, Nick Telford, Stavros Stivaros, Kate Chandler, Meenakshi Minnis, Anna Castleton, Claire Higham, Louise Hopewell, D. Gareth Evans, Anja Raams, Arjan F. Theil, Stefan Meyer, Detlev Schindler

**Affiliations:** 1 Department of Human Genetics, Biozentrum, University of Wurzburg, Am Hubland, 97074 Wurzburg, Germany; 2 Lancashire Teaching Hospitals NHS Foundation Trust, Preston, UK; 3 Oncology Cytogenetics, The Christie NHS Foundation Trust, Manchester, UK; 4 Institute of Population Health, Centre for Imaging Sciences, Faculty of Medical and Human Sciences, University of Manchester, Manchester, UK; 5 Manchester Academic Health Science Centre, Manchester, UK; 6 Department of Genetic Medicine, St Mary’s Hospital, Central Manchester Foundation Trust, Manchester, UK; 7 Department of Paediatric and Adolescent Oncology, The Christie NHS Foundation Trust, Manchester, UK; 8 Centre for Endocrinology and Diabetes, Institute of Human Development, Faculty of Medical and Human Sciences, University of Manchester, Manchester, UK; 9 Department of Molecular Genetics, Erasmus Medical Center, Rotterdam, The Netherlands; 10 Stem Cell and Leukaemia Proteomics Laboratory, Faculty of Medical and Human Sciences, University of Manchester, Manchester, UK; 11 Department of Paediatric and Adolescent Oncology, Royal Manchester Children’s Hospital, Manchester, UK; 12 Paediatric and Adolescent Oncology, Division of Cancer Sciences, University of Manchester, c/o Young Oncology Unit, Christie Hospital, Wilmslow Road, Manchester, M20 6XB UK

**Keywords:** Fanconi anemia, UV sensitivity, XPF, ERCC4, FANCQ, DNA repair

## Abstract

**Background:**

Fanconi anemia (FA) is an inherited genomic instability disorder with congenital and developmental abnormalities, bone marrow failure and predisposition to cancer early in life, and cellular sensitivity to DNA interstrand crosslinks.

**Case presentation:**

A fifty-one-year old female patient, initially diagnosed with FA in childhood on the basis of classic features and increased chromosomal breakage, and remarkable sun-sensitivity is described. She only ever had mild haematological abnormalities and no history of malignancy. To identify and characterise the genetic defect in this lady, who is one of the oldest reported FA patients, we used whole-exome sequencing for identification of causative mutations, and functionally characterized the cellular phenotype. Detection of the novel splice site mutation c.793-2A > G and the previously described missense mutation c.1765C > T (p.Arg589Trp) in *XPF/ERCC4/FANCQ* assign her as the third individual of complementation group FA-Q. Ectopic expression of wildtype, but not mutant, *XPF/ERCC4/FANCQ*, in patient-derived fibroblasts rescued cellular resistance to DNA interstrand-crosslinking agents. Patient derived FA-Q cells showed impaired nuclear excision repair capacity. However, mutated XPF/ERCC4/FANCQ protein in our patient’s cells, as in the two other patients with FA-Q, was detectable on chromatin, in contrast to XP-F cells, where missense-mutant protein failed to properly translocate to the nucleus.

**Conclusions:**

Patients with FA characteristics and UV sensitivity should be tested for mutations in *XPF/ERCC4/FANCQ*. The missense mutation p.Arg589Trp was previously detected in patients diagnosed with Xeroderma pigmentosum or Cockayne syndrome. Hence, phenotypic manifestations associated with this *XPF/ERCC4/ FANCQ* mutation are highly variable.

**Electronic supplementary material:**

The online version of this article (10.1186/s12881-018-0520-1) contains supplementary material, which is available to authorized users.

## Background

Fanconi anemia (FA) is a rare inherited genomic instability disorder with remarkable clinical and genetic heterogeneity. Whilst variable, typical features include developmental anomalies and malformations, most commonly growth retardation, cutaneous pigment displacement and radial ray defects. FA features also comprise early-onset bone marrow failure and cancer predisposition, specifically for acute myelogenous leukemia and head and neck squamous cell carcinoma [1, 2]. Causative mutations in any one of 22 FA genes (*FANCA*, -*B*, -*C*, -*D1*, -*D2*, -*E*, -*F*, -*G*, -*I*, -*J*, -*L*, -*M*, -*N*, -*O*, -*P*, -*Q, -R, -S, -T, -U* *-V-and W)* have been reported, whose corresponding proteins function together in a replication-dependent DNA interstrand crosslink (ICL) repair pathway [3, 4].

XPF, alias ERCC4 or FANCQ is the catalytic subunit of a heterodimer with ERCC1 that forms a structure-specific DNA repair endonuclease on the SLX4/FANCP scaffold. In the FA/BRCA pathway its activity is responsible for the nucleolytic strand incision 5′ to ICLs, which is a prerequisite for unhooking of a crosslink [5]. XPF/ERCC4/FANCQ also acts in the nucleotide excision repair (NER) pathway, independently of SLX4 [6], to remove *intrastrand* helix-distorting lesions from DNA, such as UV light-induced photoadducts. In fact, *XPF/ERCC4/FANCQ* mutations have mainly been identified in patients with Xeroderma pigmentosum (XP) of complementation group F (XP-F) [5, 7], and later in an individual with the XFE progeroid syndrome [8]. Recently, in two individuals (FA104 and 1333) mutations in *XPF/ERCC4/FANCQ* have been found to cause classic FA [9]. Clinical features of XP and Cockayne syndrome (CS) were described in FA due to *XPF/ERCC4/FANCQ* mutations in another patient (XPCS1CD), and reconciled clinically impaired ICL repair with deficient NER [10].

Here we present one of the oldest reported individuals to be affected by FA, whom we identified as the third FA-Q patient due to bi-allelic *XPF/ERCC4/FANCQ* mutations. As the missense mutation p.Arg589Trp here implicates FA, opposed to XP or CS in other cases, we investigate and discuss biological and clinical implications.

## Case presentation

### Clinical course

**Fig. 1:**
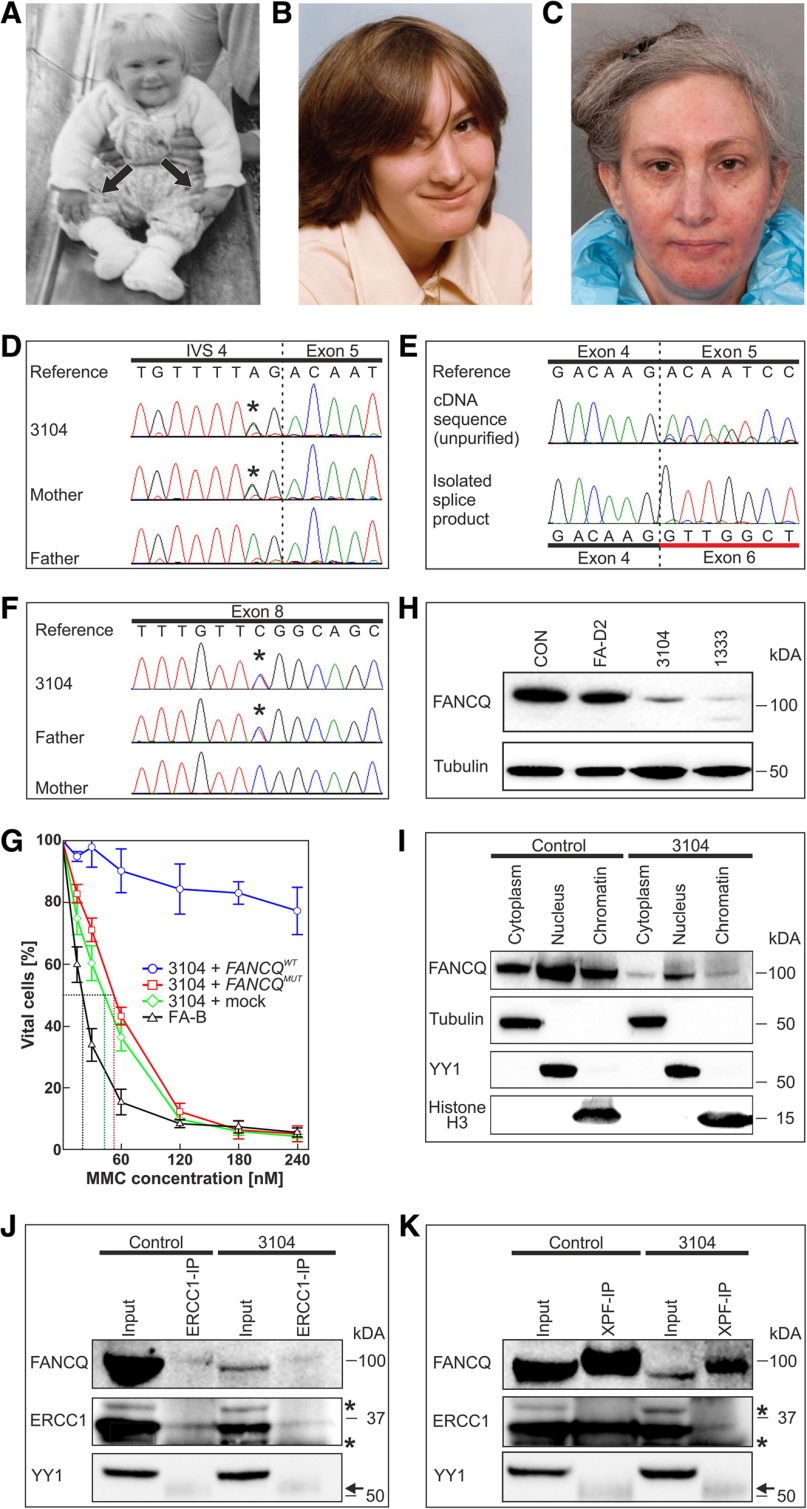
Portraits of FA-Q patient 3104 at different age, and characterization of her genetic and cellular defects. **a:** Typical FA puppet-like face, small head, light brown hair, absence of the right and severe hypoplasia of the left thumb (arrows) at about 15 months. **b:** High slanting eye lids and beginning freckling of the face at age 12 years. **c:** Low-set auricles, early hair graying and actinic keratosis-like skin changes with erythema, atrophy and patchy pigmentation in her fifth decade. **d:** A heterozygous substitution of adenine with guanine (asterisk) 2 bp upstream of exon 5 in the *XPF/ ERCC4*/*FANCQ* gene was maternally inherited. **e**: The mutation shown in **(d)** leads to aberrant splicing (upper panel). Gel extraction and sequence analysis of a major splice product reveals skipping of exon 5 (lower panel). **f**: The second heterozygous *FANCQ* mutation c.1765C > T (asterisk) is located in exon 8 and was paternally inherited. **g:** 3104 fibroblasts transduced with mock vector (green line) or with vector containing *XPF/ ERCC4*/*FANCQ* with the missense mutation c.1765C > T (red line) are MMC-sensitive, though not quite to the degree of fibroblasts from an FA-B patient serving as a control (black line). Complementation of 3104 fibroblasts with wildtype *FANCQ* (*FANCQ*^*WT*^, blue line) restores MMC resistance. Error bars designate SDs of three experiments. LC_50_ levels are indicated by dotted lines of corresponding colors; they equal 42.6 ± 5.7 nM for 3104 + mock, 52.8 ± 2.7 nM for 3104 + *FANCQ*^*MUT*^ and 20.7 ± 2.4 nM for FA-B fibroblasts. The survival rates of 3104 + *FANCQ*^*MUT*^ and 3104 + mock are not significantly different, however the proportions for all concentrations (except 0) of these curves and 3104 + FANCQ^WT^ are different with *p* < 0.001. **h:** Immunoblot using fibroblast extracts from the FA-Q cell line 3104 and of the previously reported FA-Q cell line 1333 [9] with different *FANCQ* missense mutations showing immunoreactive residual FANCQ protein of normal size but reduced abundance in comparison to a normal (CON) and a FANCD2-deficient (FA-D2) control. One thousand three hundred thirty-three reveals an additional FANCQ band resulting from a truncating mutation on the second allele. Loading control: tubulin. **i:** Cell fractionation demonstrates residual mutant FANCQ protein in 3104 cells detectable in the chromatin fraction. **j** and **k:** Normal ERCC1-XPF interactions in 3104 transformed fibroblasts. ERCC1 (**j**) or XPF (**k**) were immunoprecipitated with antibodies against ERCC1 or XPF, respectively. Unspecific bands are marked by asterisks while the visible antibody heavy chain is indicated by an arrow

The diagnosis of FA in a 51-year-old woman (at the time of this report, patient 3104) was made when she presented at the age of seven with thrombocytopenia and bilateral radial ray abnormalities including right thumb absence and severe left hypoplasia (Fig. 1a), and increased sensitivity to DNA interstrand-crosslinking agents of her cells (data no longer available). Other features of FA included mild facial anomalies (Fig. 1a−c), short stature, microcephaly and skin pigmentation changes. The patient is the younger of two sisters of an unrelated White British couple, both over 70 and well, with an unremarkable past medical and family history. The patient is married and worked for a long time in administrative and secretarial positions. During her adult life borderline thrombocytopenia and macrocytosis persisted. Further details with respect to clinical manifestations and management are provided in Table 1. Since she was referred to our service in her fifth decade, her hypo-cellular bone marrow remained without features of dysplasia or clonal cytogenetic aberrations (current platelet count 117 × 10^9^/L, MCV 109 fl). Notably, she reported to burn very easily in the sun, and avoided sun exposure all her life. She developed so far neither bone marrow failure nor malignancy.

**Table 1:** Clinical manifestation of affected systems and clinical management in the FA-Q patient

Affected System	Clinical manifestations	Management
Growth	Low birthweight 2010 g; Short stature; Microcephaly	
Skeletal abnormalities	Right thumb absent Left thumb hypoplastic Hip dislocation Tibia torsion	Pollicizations at age 3 and 4 Conservative Internal rotation osteotomy age 25
Gastrointestinal	Ectopic anus Fatty liver with persistently elevated liver enzymes	Monitoring
Urogenital	Crossed kidney fusion ectopia, both kidneys on the right	Surveillance of kidney function only
Haematological	Thrombocytopenia with macrocytosis Mild bone marrow hypoplasia without evidence of clonal chromosomal aberrations	Surveillance only
Endocrine and reproductive health	Diabetes Type 1, age 42 No children Menopause age 29	Low dose insulin (~0.2 U/kg/ day) Hormone replacement therapy since age 32
Skin	Café-au-lait spots Lifelong sun sensitivity with severe sunburn and blistering after minimal sun exposure	Meticulous sun avoidance and skin protection
Hearing	Sensorineural hearing loss increasingly relevant age 45	Hearing aid
Brain	Cerebral and cerebellar atrophy Small pituitary gland Microangiopathic white matter signal changes	

### Cell culture

Cyclosporine A was used to establish Epstein-Barr virus (EBV)-transformed lymphoblastoid cell lines (LCL) [11]. Peripheral blood lymphoblasts were grown in RPMI 1640 medium supplemented with GlutaMAX (Gibco) and 15% fetal bovine serum (FBS). Primary fibroblasts of the patient concerned were cultured in Amniopan medium (PAN Biotech), while SV40 large T antigen-immortalized fibroblasts were maintained in MEM with GlutaMAX and 10% FBS. For retrovirally transduced fibroblasts 10% Tet System Approved FBS (Clontech Laboratories) was used. All cultures were maintained in incubators with 5% CO_2_. Cells were exposed to 40 ng/μl mitomycin C (MMC) for 16 h prior to immunofluorescence analysis of RAD51 foci, immunoblotting or cell fractionation studies. Cell lines from disease and normal controls were maintained as previously reported [9].

### Chromosome studies

Cytogenetic assays were performed on whole blood cultures to assess spontaneous and MMC-induced breakage rates. MMC at indicated final concentrations was added to blood samples as a G_0_ pulse before culture initiation. After 1 h the cells were washed and transferred to fresh complete RPMI 1640 medium. Lymphocytes were stimulated with phytohemagglutinin (PHA). The cultures were incubated at 37 °C and harvested after 72 h. Chromosome preparations were made by the air drying method [12]. Solid (Giemsa)-stained metaphases were examined for chromatid and chromosome type damage, and the results were compared to age and sex-matched normal controls.

### Cell cycle analysis

PHA-stimulated lymphocytes or primary fibroblasts were cultured untreated for 72 h or continuously exposed to MMC. Mono- or bivariate (BrdU-Hoechst 33,258/Ethidium bromide) cell cycle analysis was performed on a triple-laser-equipped flow cytometer (LSRII, BD Biosciences). Data were analyzed using the MPLUS AV software package (Phoenix Flow Systems) [13].

### Nucleic acid isolation and cDNA synthesis

A modified salting-out technique [14] or the GeneJET Genomic DNA Purification Kit (Thermo Fisher) were used for isolation of genomic DNA (gDNA). For total RNA isolation we employed the High Pure RNA Isolation Kit (Roche) while transcription into cDNA was performed by SuperScript Reverse Transcriptase (Invitrogen).

### Quantitative PCR

For relative quantification of mRNA expression levels, allele-specific primers were designed (WT for: 5’-GACGCAGAGCTAACCTTTGTT**C**-3′; c.1765C > T MUT for: 5′- GACGCAGAGCTAACCTTTGTT**T**-3′; WT/MUT rev: 5′- GTTCCTCAGTTGAACCTCCGTA-3′). For the detection of exon 5 skipping due to c.793-2A > G primer sequences were as follows: WT for: 5′- TCTGGAATCTCTGAGAGCAACG-3′, WT rev: 5′- AACATCGAGGTGCTGGAGTC-3′, MUT for: 5′- ATAACCCATCGCTTGAAGTGGA-3′, MUT rev: 5′- CAAGAA\ACAGCCAACCTTGTCA-3′. The PCR reaction was performed with HOT FIREPol® EvaGreen® qPCR Mix Plus (Solis BioDyne) on an ABI ViiaTM 7 System (Applied BiosystemsTM). Evaluation of melting curves and amplification plots, and relative quantification (RQ) was done with the QuantStudioTM Real-Time PCR Software v1.2.4 (Applied BiosystemsTM) using the ΔΔCt method. Each sample was analyzed in technical triplets.

### Retroviral complementation

Transduction with different retroviral vectors containing *XPF/ERCC4/FANCQ* cDNA constructs or mock was performed according to standard protocols [15, 16]. Transduced immortalized fibroblasts were analyzed for their sensitivity. Aliquots of 20,000 cells per well were seeded in 6-well culture plates and grown at the indicated concentrations of MMC. After eight days cell viability was determined by image cytometry using VitaBright-48 and propidium iodide staining on a NucleoCounter NC-250 instrument (ChemoMetec A/S).

### Lymphoblast survival

LCLs were grown in T25 cell culture flasks at concentrations of 0–1000 nM MMC for eight days. Cell viability was analyzed by propidium iodide exclusion and assayed by flow cytometry.

### Immunofluorescence

Nuclear RAD51 focus formation was analyzed in fibroblasts grown on glass chamber slides (Sarstedt). Cells were washed with PBS and subsequently fixed in 4% (vol/vol) paraformaldehyde in PBS for 15 min at room temperature. Thereafter they were exposed to ice-cold 100% methanol and kept on ice for 30 min. Blocking of non-specific antibody binding sites was accomplished with 20% (vol/vol) FBS in PBS for 30 min at room temperature. Rabbit anti-RAD51 (1:800; ab63801, Abcam) served as primary antibody, to be detected by Alexa 594-conjugated anti-rabbit secondary antibody (1:2000; A11037, Molecular Probes/Life Technologies). ProLong Gold Antifade Mountant with DAPI (Thermo Fisher) was used for counter-staining and as mounting medium. Foci-positive cells (>5 foci/nucleus) were scored by fluorescence microscopy (Axiovert 40C, Zeiss).

### Immunoblot analysis

Aliquots of 40 μg whole protein extracts from cultured cells were loaded on NuPAGE Novex 7% Tris-Acetate protein gels (Invitrogen). Electrophoresis was carried out over night at 70 V with constant cooling. Proteins were transferred using a dry blotting system (iBlot 2, Life Technologies). FANCD2 isoform immunodetection was carried out using primary anti-FANCD2 antibody (1:500; sc-20,022, Santa Cruz) and secondary anti-mouse IgG-HRP (sc-2005, Santa Cruz) diluted 1:3000. FANCJ (1:1000; NB100–416, Novus) or vinculin (1:10,000; ab129002, Abcam) served as loading controls. For visualization of ERCC4/XPF/FANCQ whole protein extracts were separated on NuPAGE 4–12% Bis-Tris gels (Invitrogen). Anti-XPF (1:200; ab17798, Abcam) was used for immunodetection while Anti-Tubulin (1:1000; ab44928, Abcam) was employed as loading control. Luminescence was generated by chemical reaction with a standard ECL reagent (Millipore Corporation).

### Subcellular fractionation

The NE-PER Nuclear and Cytoplasmic Extraction Kit (Thermo Fisher) was used for extraction of cytoplasmic proteins while nuclear and chromatin-associated proteins were isolated by the Subcellular Protein Fractionation Kit (Thermo Fisher). SDS-PAGE was run on NuPAGE 4–12% Bis-Tris gels (Invitrogen). For XPF, YY1, tubulin and histone H3 immunodetection we employed Anti-XPF (1:200; ab17798, Abcam), Anti-Tubulin (1:1000; ab44928, Abcam), Anti-YY1 (1:5000; ab199998, Abcam) and Anti-Histone H3 (1:800; ab1791, Abcam) antibodies. Secondary antibodies were as above.

### Co-Immunoprecipitation

Cell pellets were resuspended in 200 μl lysis buffer (20 mM Tris-HCl pH 7.4, 10 mM KCl, 140 mM NaCl, 1 mM EDTA, 0.2 mM EGTA, 0,5% Triton X-100, 5% Glycerol, 0.1 mM PMSF, 0.5 mM DTT, Halt Protease/Phosphatase Inhibitor Cocktail 1:50) and lysed by sonication. After centrifugation (14,000×g for 10 min at 4 °C) and lysate pre-clearing, 500 μl cell lysate with 1 mg/ml cell total protein was incubated with the primary antibody (ERCC1: ab2356, Abcam, 1:150; XPF: ab76948, Abcam, 4 μg/mg total protein in the lysate) at 4 °C overnight. 50 μl of pre-washed Protein G Dynabeads (Thermo Scientific) was added. After incubation for 2 h at 4 °C, the beads were washed five times using 500 μl washing buffer (20 mM Tris-HCl pH 7.4, 10 mM KCl, 140 mM NaCl, 1 mM EDTA, 0.2 mM EGTA, 0.1% Triton X-100, 5% Glycerol, 0.1 mM PMSF, 0.5 mM DTT, Halt Protease/Phosphatase Inhibitor Cocktail 1:100). Beads were taken up in 20 μl elution buffer, 4× LDS sample buffer and 10× reducing agent. Following incubation for 10 min at 70 °C beads were collected and the supernatant was loaded on a 4–12% Bis-Tris gel. Immunodetection was carried out using anti-XPF (1:200; ab17798, Abcam) or anti-ERCC1 (1:100; sc-17,809, Santa Cruz) primary antibodies. YY1 (1:5000; ab199998, Abcam) served as loading control.

### UV-C survival

Aliquots of 5000 fibroblasts per well were seeded in 6-well plates. After two days cells were washed with PBS and irradiated at the indicated doses in quadruplicate (0 J/m^2^) or triplicate (others) using an UV-C germicidal lamp (254 nm; Philips). An UVX Digital Radiometer (Serial No. E27846, UVP) was used to quantify the exact UV dose. Control cells were UV irradiated simultaneously to serve as an internal control. After another five days, before the non-irradiated cultures reached confluency, cells were pulse-labeled with [methyl-^3^H]-thymidine (40–60 Ci/mmol; 5 μCi/ml; Amersham Biosciences)-containing medium for 3 h, washed with PBS and chased for 15 min in medium without ^3^H–thymidine. Finally cells were lysed in 0.25 M NaOH. Each lysate was counted with 7.5 ml Hionic Fluor scintillation fluid in a liquid scintillation counter (Packard) for 10 min. The results obtained from irradiated plates were expressed as percentages of non-irradiated plates (set 100%) and plotted [17].

### UV-C-induced UDS and RRS

Unscheduled DNA synthesis (UDS) and recovery of RNA synthesis (RRS) experiments after UV-C irradiation were done with mixed cell populations [7]. C5RO wildtype (WT) fibroblasts were incubated and preloaded with polystyrene beads of 2 μm diameter for three days, then mixed with the patient (3104 or 1333) fibroblasts and seeded on coverslips. Two days later adherent cells were washed with PBS and UV-C irradiated at 16 J/m^2^. Thereafter they were incubated for 3 h in medium containing 0.1 μM 5-ethynyl-2′-deoxyuridine (EdU; Invitrogen). Afterwards they were washed with PBS, incubated with medium without EdU for 15 min, washed with PBS, fixed with 3.7% formaldehyde in PBS containing 0.5% Triton for 15 min and washed two times with PBS again. The cells were incubated for 30 min with fluorescent dye-coupling buffer containing 10 mM CuSO_4_ and Alexa Fluor 594 azide (Qlick-iTTM; Invitrogen). After washing with PBS, cells were mounted in Vectashield Antifade Mounting Medium with DAPI (Vector Laboratories). For RRS studies cells were treated identically as for UDS experiments, but 16 h after irradiation incubated with culture medium containing 0.1 μM 5-ethynyl-uridine (EU) for 2 h.

Visual light distinguished WT cells that contained beads in their cytoplasm from patient cells that did not. On micrographs DAPI is the blue signal that stains nuclei. Red is the UDS (or RRS) signal; it is much brighter in WT than in patient cells. UDS and RRS levels are expressed as the average fluorescence intensity in the nucleus of patient vs. WT cells (set at 100%).

### Statistics

An unpaired two-tailed Student’s t test was used to compare the results of qPCR analyses. A *p* value less than 0.05 was considered significant; *p < 0.05, **p < 0.01, ***p < 0.001.

### Results

Chromosome breakage studies from peripheral lymphocyte culture of the patient confirmed FA at ages 7, 33 and 49, and demonstrated increased levels of spontaneous damage, and in response to MMC (Additional file 1: Table S1, results of the analysis carried out age 7 are no longer available). In primary fibroblast cultures a spontaneous and MMC-induced G2-phase arrest was observed (Additional file 1: Figure S1A). Also peripheral blood lymphocyte cultures showed G2-phase accumulation within the FA-range, excluding mosaicism in the hematopoietic system (Additional file 1: Figure S1B). Functional analysis of FANCD2 ubiquitination in 3104 fibroblasts demonstrated both FANCD2 isoforms (Additional file 1: Figure S1C), indicating a mutation in an FA gene downstream of those encoding the FA core- and FANCD2/FANCI-complex. RAD51 foci developed normally after exposure to MMC in both 3104 fibroblasts and lymphoblasts (Additional file 1: Figure S1D), excluding the post-FANCD2 groups FA-D1, -N and -O, −R, –S, and -U. WES identified two mutations in the *XPF/ERCC4/FANCQ* gene, which were confirmed by Sanger sequencing. A single nucleotide substitution located two base pair upstream of exon 5 (c.793-2A > G) was detected on the maternal allele, affecting a canonical splice acceptor (Fig. 1d). cDNA sequencing verified aberrant splicing. A major splice product revealed exon 5 skipping (Fig. 1e). This event is predicted to result in premature termination of translation (p.Thr265Valfs*13). The second change in the *XPF/ERCC4/FANCQ* sequence is the missense mutation c.1765C > T in exon 8 (Fig. 1f) on the paternal allele, which is listed on the ExAC-Browser with a minor allele frequency of 0.0066%. It substitutes a highly conserved amino acid residue (p.Arg589Trp) in the SF2 helicase-like domain. This mutation has previously been reported in two XP-F patients (XP24BR and XP32BR) [18], one patient with XP with neurodegeneration (AS871) [5], and an individual showing an intermediate XP/CS phenotype and features of FA (XPCS1CD) [10]. Expression of both mutations in mRNA was re-confirmed by qPCR (Additional file 1: Figures S1E and F). Complementation of 3104 fibroblasts by wildtype, but not Arg589Trp-mutant *XPF/ERCC4/FANCQ* rescued MMC resistance, providing evidence that the mutations of *XPF/ERCC4/FANCQ* cause the cellular FA phenotype (Fig. 1g). Premature termination of translation due to exon 5 skipping would result in a truncated protein of 31.4 kDa. However, on XPF/ERCC4/FANCQ immunoblot analysis of 3104 fibroblasts only one low signal intensity band of approximately 100 kDa was detected (Fig. 1h), suggesting the presence of residual mutant XPF/ERCC4/FANCQ protein of normal size but reduced abundance. Dilution studies demonstrated that XPF/ERCC4/FANCQ residual protein with the Arg589Trp mutation is present at approximately a 1:15th the level of wildtype XPF/ERCC4/FANCQ protein (Additional file 1: Figure S1G). However, it is difficult to tell the individual contributions of mutant transcript and protein instability to this reduction. Fractionation studies of 3104 fibroblasts showed that residual full-length XPF/ERCC4/FANCQ protein was detectable in the nucleus (Fig. 1i), while in cells from XP-F or XFE patients the mutant XPF/ERCC4/FANCQ appears to be mislocated and not to be part of the XPF-ERCC1 complex, presumably as a consequence of XPF protein misfolding [19].

**Fig. 2:**
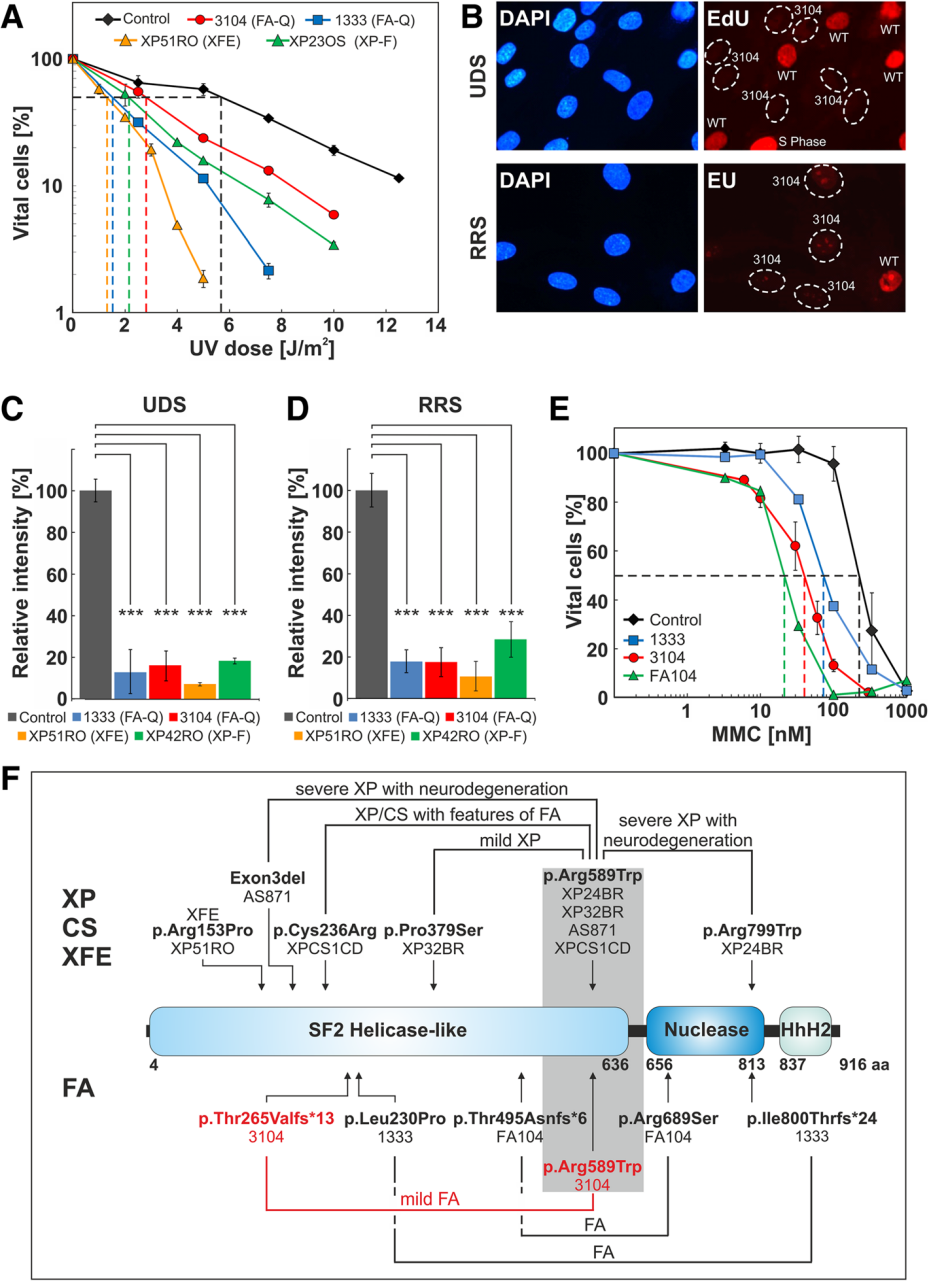
*ERCC4*/*XPF/FANCQ* (missense) mutations and their effects. **a:** Dose-response curves of FA-Q fibroblasts 3104 (red) compared with previously reported 1333 (blue), XFE (XP51RO; orange) and XP-F fibroblasts (XP23OS; green) reveal different degrees of UV-C sensitivity on survival compared with normal control fibroblasts (black) (means ± SEMs). LC_50_ levels are indicated by the dashed lines of corresponding colors; they equal 5.7 J/m^2^ for control cells, 2.8 J/m^2^ for 3104, 1.5 J/m^2^ for 1333, 2.2 J/m^2^ for XP23OS and 1.3 J/m^2^ for XP51RO. All mentioned cells represent primary fibroblasts. **b:** NER activity of 3104 FA-Q primary fibroblasts assayed ex vivo. UDS (upper panel) was measured by incorporation of 5-ethynyl-deoxyuridine (EdU), RRS (lower panel) by incorporation of 5-ethynyl-uridine (EU). Mixed-in wildtype (WT) fibroblasts preloaded with polystyrene beads (not depicted) served as internal control. DAPI as nuclear counterstain for all cells. UDS and RRS signals were quantified from 20 to 40 random nuclei. **c:** UDS levels of control fibroblasts (grey) compared to FA-Q 3104 (red), 1333 (blue), XFE (orange) and XP-F (green) fibroblasts (means ± SEMs, brackets indicates levels os significance). **d:** RRS levels of control fibroblasts (grey) compared to FA-Q 3104 (red), 1333 (blue), XFE (orange) and XP-F (green) fibroblasts (means ± SEMs, brackets indicates levels os significance). **e:** Dose-response curves of 3104 (red; means ± SEMs) and two previously reported FA-Q lymphoblast lines (FA104, green; 1333, blue) exposed to MMC show different degrees of sensitivity compared to normal control lymphoblasts (black). LC_50_ levels are indicated by dashed lines of corresponding colors and equal 210 nM MMC for the control, 80 nM MMC for 1333, 42 nM MMC for 3104 and 24 nM MMC for FA104. **f:** Combinations (brackets) of the biallelic mutations in all XP-F, CS, XPCS(FA) and XFE patients with p.Arg589Trp (shaded) (above) and of the biallelic mutations in the three reported FA-Q patients (below) are depicted relative to location in the ERCC4/XPF/FANCQ protein domain structure [14]. The predicted mutational effects on the protein level are shown in bold letters together with the corresponding patient designations listed below. Individuals with XP-F include XP24BR, XP32BRand AS871, with XFE include XP51RO, with XPCS(FA) include XPCS1CD, and with FA-Q include 3104, FA104 and 1333 (3104 highlighted in red). The phenotypic severity of disease in patients with XP, CS, XPCS(FA), or FA-Q carrying one heterozygous p.Arg589Trp mutation is indicated above the brackets

In addition to ICL repair, NER activity was also impaired in 3104 fibroblasts. Cell survival after UV-C irradiation was reduced (Fig. 2a) (LC_50_ = 2.8 J/m^2^), as it was the case in fibroblasts from an XP-F patient with known UV sensitivity and a mild clinical phenotype (LC_50_ = 2.2 J/m^2^) [20], and FA-Q 1333 fibroblasts as previously reported [9]. Fibroblasts from the unique XFE patient [8] showed much higher sensitivity (LC_50_ = 1.3 J/m^2^). Both global genome NER (GG-NER) and transcription-coupled NER (TC-NER) were affected in cells from patient 3104. Unscheduled DNA synthesis (UDS) and recovery of RNA synthesis (RRS) in 3104 fibroblasts after UV irradiation were more than 80% decreased compared to controls (Fig. 2b-d), to similar levels as for FA1333 fibroblasts was previously reported [9] and is shown here again (Fig. 2c and d). Fibroblasts from an XP-F patient with mild clinical UV-light sensitivity (XP42RO) [7] also exhibited comparable reduction of UDS and RRS rates, whereas fibroblasts from the XPE patient (XP51RO) had even lower activity (Fig. 2c and d). In contrast, 3104 cells were more MMC-sensitive than 1333 cells (Fig. 2e).

## Discussion and conclusion

Long-term observations and genotype/phenotype correlations in rare variants of DNA-repair disorders unlock a potential for understanding gene function and specific effects in the DNA-damage response network. Here we describe one of the oldest FA patients reported, and the first case who gave a strong history of UV-light sensitivity, which was confirmed on the cellular level. Hence, UV sensitivity can be a feature of FA-Q, and the patient’s perception might be the clinical diagnostic clue for complementation group assignment and mutation detection in unclassified FA patients. From a hematological perspective she has done remarkably well, particularly as she has radial ray malformations which are often associated with early hematological problems [21].

The Arg589Trp mutation observed in individual 3104 appears to be associated with mislocalization of XPF/FANCQ/ERCC4 protein in XP-F cells, where missense-mutant protein failed to properly translocate to the nucleus [19]. Therefore, the DNA repair defect in XP with this mutation is at least in part likely due to mislocalized protein that still interacts with ERCC1, irrespective of its subcellular localization [19]. We performed co-immunoprecipitation experiments using anti-ERCC1 or anti-XPF antibodies. Both ways, we were able to confirm the interaction in 3104 fibroblasts. In contrast to the literature [19], residual mutant XPF/FANCQ/ERCC4 of patient 3104 reported here was detectable with chromatin, as previously described for both other reported FA-Q-associated mutations Leu230Pro and Arg689Ser [9], where an interaction of missense-mutant XPF/FANCQ/ERCC4 proteins with the scaffolding protein SLX4/FANCP and its dimeric partner ERCC1 was identified. Hence, the loss of mutant protein interactions that is critical for the FA-phenotype in FA-Q patients seems unlikely, as Arg589Trp should have the same effect also in XP-F patients. Additional factors modulating the clinical phenotype, which are relevant for FA-Q patients could include a mechanism allowing a proportion of mutant protein to escape misfolding. It will be important to determine if natural chaperones play a role in rescuing XPF/FANCQ/ERCC4 conformational defects, as in other protein-misfolding diseases, to pave the way clinically from XP or CS to FA [21]. The concept of manipulating XP, CS, or XFE therapeutically by targeting nuclear re-localization of mutant XPF-ERCC1 as previously suggested [19], e.g. by pharmaceutical chaperones, is questioned by our study, as the problem with ICL repair may remain. The reverse, however, targeting interactions or functions of XPF/FANCQ/ERCC4, a key protein at the intersection of DNA repair pathways, could disable NER and ICL repair functions at the same time and might represent a promising approach to overcome chemo-resistance in malignant disease [22].

In the two other reported FA-Q patients FA104 and 1333 both second *XPF/FANCQ/ERCC4* mutations are null alleles [9]. In XPCS1CD the missense mutation p.Cys236Arg confers the XPCS phenotype by conveying insufficient NER activity, but fails to provide endonuclease activity for ICL removal, being a functional null allele in this respect [10]. In the FA patient presented here, the second mutation affects a canonical splice acceptor site (c.793-2A > G) resulting in exon 5 skipping, frameshift and predicted protein truncation. We show that the mutant transcript is relatively unstable and can be stabilized with cycloheximide (Additional file 1: Figure S1F). Moreover, we demonstrate that residual abridged XPF/FANCQ/ERCC4 protein is absent (Fig. 1h) such that this splice site mutation can be regarded as a null allele. In addition there is no cryptic splice acceptor nearby that could restore the reading frame. Pathogenicity of the splice mutation is underscored by the fact that there is neither a listed transcript isoform lacking exon 5 sequence nor a reported alternative *ERCC4/XPF/FANCQ* splicing product that involves skipping of exon 5. Our findings imply that the common basis of all FA-Q cases to date is compound heterozygosity for one null and one hypomorphic missense mutation in *XPF/FANCQ/ERCC4*, which facilitates the retention of residual mutant protein capable of chromatin relocation and the provision of NER activity [9], possibly providing the genetic basis for long-term survival of certain FA-Q patients.

## Additional file

Additional file 1: Figure S1. and **Table S1.** (DOCX 4108 kb)
