# Supplementary material for: Fanconi anemia with sun-sensitivity caused by a Xeroderma pigmentosum-associated missense mutation in XPF
Source: BMC Med Genet. 2018 Jan 11;19:7. doi: 10.1186/s12881-018-0520-1 (PMC5765604; doi:10.1186/s12881-018-0520-1)
Supplement: Additional file 1: Figure S1. — and Table S1. (DOCX 4108 kb) [file 12881_2018_520_MOESM1_ESM.docx]

**Fanconi anemia with sun-sensitivity caused by a Xeroderma pigmentosum associated missense mutation in *XPF –* case report of the third FA-Q patient**

Popp *et al*

**SUPPLEMENTARY MATERIAL**

**Supplementary table S2:** Chromosomal breakage* of patient 3104 at different age

| **MMC concentration [nM]** | **Patient breakage rate**  **[breaks per metaphase]** | | **Normal control range**  **[breaks per metaphase]** |
| --- | --- | --- | --- |
|  | at 33 years | at 49 years |  |
| 0 | 0.07–0.15 | 0.08 | ≤ 0.02 |
| 30 | n.d. | 0.59 | ≤ 0.03 |
| 60 | n.d. | 1.13 | ≤ 0.05 |
| 100 | 1.4–1.5 | n. d | ≤ 0.06 |

*Spontaneous and MMC-induced sensitivity was assessed on phytohaemagglutinin-stimulated peripheral blood cultures and compared to an age and sex-matched controls.

**SUPPLEMENTARY FIGURE S1**


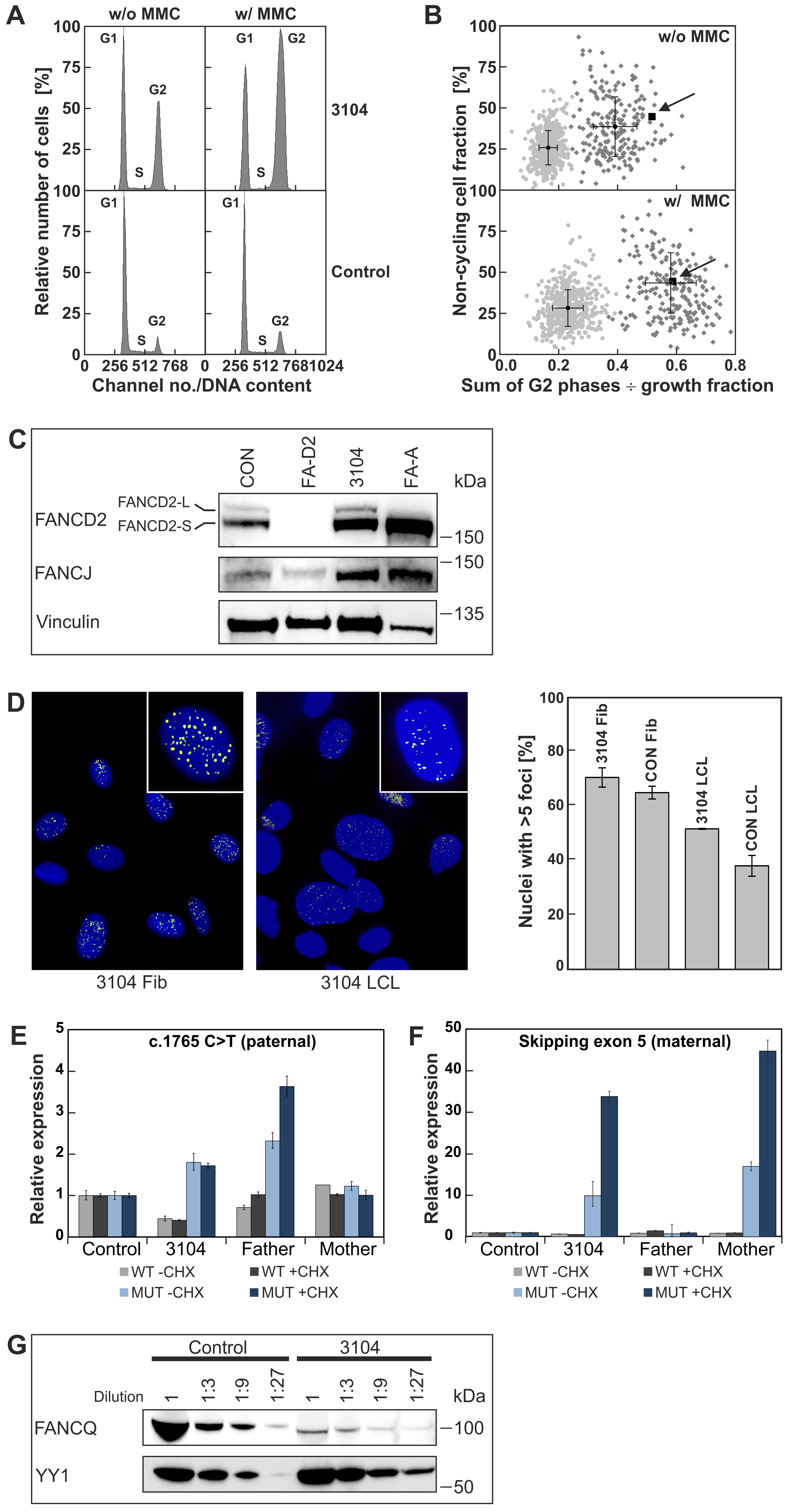


**Supplementary figure S1:** Cellular studies to confirm the diagnosis of FA in individual 3104 and to assign her to a downstream FA complementation group. **A:** 3104 primary fibroblasts were analyzed for their hypersensitivity to MMC by flow cytometry and revealed a high degree of both spontaneous (upper left panel) and MMC-induced (upper right panel) G2-phase arrest. A normal control is shown by comparison (lower panels). Cells accumulated in G2 are indicated by arrows and quantitated. **B:** G2-phase accumulation was confirmed in peripheral blood lymphocytes. The G2 phase over growth fraction ratio of individual 3104 (black square, arrow) clearly lies within the range of other FA patients (dark grey squares), distinct from normal controls (light grey dots). Error bars denote SDs. Cells were analyzed without (upper panel) and after (lower panel) exposure to MMC. **C:** FANCD2 immunoblot analysis detected both isoforms (FANCD2-L and FANCD2-S), assigning patient 3104 to a downstream complementation group. FANCJ and vinculin served as loading controls. **D:** RAD51 foci formation proficient in both 3104 fibroblasts (left panel) and LCLs (middle panel). The fractions of nuclei with >5 foci were comparable to a normal control (CON) (right panel). **E** and **F:** Allele specific expression analysis with RT-PCR in patient and parent compared to control cells for paternal allele c.1765C>T, and maternal allele with skipped exon 5 . **G:** Immunoblot detection of XPF protein in contol cells compared with patient derived 3104 cells. YY1 as loading control.
